# Identification of phloem-specific proteinaSEOus structure heterogeneity in sieve element of *Populus trichocarpa*

**DOI:** 10.1186/s12870-025-06439-4

**Published:** 2025-04-10

**Authors:** Karolina Kułak, Anna Samelak-Czajka, Malgorzata Marszalek-Zenczak, Kornel M. Michalak, Magdalena Trybus, Julia Minicka, Paulina Jackowiak, Agnieszka Bagniewska-Zadworna

**Affiliations:** 1 Department of General Botany, Institute of Experimental Biology, Faculty of Biology, Adam Mickiewicz University, Uniwersytetu Poznanskiego 6, Poznan, 61-614 Poland; 2 Laboratory of Single Cell Analyses, Institute of Bioorganic Chemistry, Polish Academy of Sciences, Noskowskiego 12/14, Poznan, 61-704 Poland; 3 Department of Virology and Bacteriology, Institute of Plant Protection – National Research Institute, Wegorka 20, Poznan, 60-318 Poland

**Keywords:** Phloem, Sieve element, P-protein, SEO, SEOR, *Populus trichocarpa*

## Abstract

Phloem, an exceptional plant vascular tissue, facilitates the transport of photoassimilates, RNAs, and other signaling substances from the leaves to the roots throughout the plant. Among the specialized phloem cells are the conductive sieve elements (SEs), which are unique in that they remain alive despite lacking several cell organelles, including the nucleus, plastids, and most mitochondria. These SEs contain a specific proteinaceous structure composed of phloem-specific proteins (P-proteins), whose function is not yet fully understood. Various P-proteins have been characterized in broad range of model species, including *Arabidopsis thaliana*, and reported in Fabaceae and Cucurbitaceae plants. To date, only one P-protein has been identified in the model tree species *Populus trichocarpa*. Given the presence of multiple P-protein encoding genes across numerous plant species, we hypothesized the existence of multiple such genes in the *Populus* genome. Our genomic analysis uncovered 12 genes being potential orthologues to one of *A. thaliana* P-protein* – SEOR* (*sieve element occlusion-related*) genes, which may contribute to the proteinaceous structures observed in differentiating sieve elements. Our transcriptomic and proteomic analyses confirmed the expression of at least seven of these genes, indicating that the protein structure visible in mature sieve elements in *P. trichocarpa* may be heterogeneous.

**Supplementary Information:**

The online version contains supplementary material available at 10.1186/s12870-025-06439-4.

## Background

One of the principal adaptations plants have developed during their evolution to thrive in terrestrial environments is the capability to transport assimilates and signaling molecules throughout their structure, a function primarily facilitated by phloem tissue. This component of the plant vascular system is engaged in the transport of photoassimilates, RNAs, and other signaling molecules from leaves to all parts of the plant [1]. Phloem is characterized by a high degree of cell specialization and heterogeneity, attributed to the fact that not all phloem cells serve a conductive function. This tissue includes conductive sieve elements (SEs), companion cells, phloem parenchyma involved in radial transport and storage, and dead fiber cells that provide mechanical support. Sieve elements are distinctive cell types that lack certain organelles such as the nucleus, plastids, and most mitochondria during differentiation, yet they remain viable [2, 3].

Most angiosperm plants generate phloem-specific proteins (P-proteins) in their SE precursor cells [1]. The initial observation of sieve element-specific protein structures, termed “slime” by Hartig in 1854, noted a specific accumulation of material on the sieve plates or across the sieve element lumen [4]. Subsequent studies confirmed the proteinaceous nature of this slime [5], leading to these proteins being designated as "P-protein" [6]. Despite investigations into P-proteins since the 1960s, their functions remain largely speculative. What is known now is these proteins recognition as aggregates detectable in angiosperm sieve elements [7] and exhibition a variety of structures across different plant species and developmental stages [8, 9]. Their potential roles have long been hypothesized [10], predominantly as mechanisms for sieve element occlusion [11–13] or as physical barriers against phloem-feeding insects or microbes [10, 13]. These hypothetical functions have been comprehensively reviewed by Noll et al. [14]. To date, the specific functions of P-proteins remain elusive, with the notable exception of forisomes in Fabaceae species, which have been demonstrated to participate in responses to certain phloem-feeding aphids [15].

These proteins are known to assemble into dense bodies during SE differentiation, dispersing in mature phloem conductive cells [16]. However, certain P-protein bodies, identified as non-dispersive P-protein bodies (NPBs), were initially only documented in dicot plants [17] before being detected in the monocot family of Zingiberaceae [18]. Intriguingly, NPBs were first thought to be degraded nuclear remnants [19, 20] until ultrastructural studies confirmed their protein aggregation nature [21, 22]. P-protein synthesis commences prior to sieve element enucleation [23], resulting in electron-dense structures initially described as granular, fibrillar, or tubular [6, 24, 25]. These structures have been observed within the same cell, suggesting that they represent various stages of the P-protein differentiation process [8]. During sieve element maturation, some P-proteins form into large bodies, which become smaller and relocate to the cell periphery during selective autophagy events in SE development, taking their position in the mature SE [26]. Due to their location and potential functions, these proteins have been named SEO (sieve element occlusion) proteins.

Certain plant families exhibit distinct P-protein characteristics, notably the Cucurbitaceae, whose dispersive P-proteins undergo reversible, oxidative cross-linkage to form high molecular weight polymers [10]. In cucurbits, a specific phloem filament protein, PP1, is not a homolog of sieve element occlusion proteins (SEOs) [27]. This uniqueness suggests that the filaments formed by PP1 are specific to the large sieve elements of cucurbits [28]. Another well-studied group in terms of P-proteins is the Fabaceae, which feature unusual phloem protein structures called forisomes [29]. These are non-dispersive P-protein bodies that can transition into dispersed states in response to changes in local calcium concentrations [29, 30]. In *Medicago truncatula*, for example, forisomes consist of at least three SEO proteins [27], while a broader range of SEO family genes has been identified in this species [31]. The number of SEO encoding genes varies significantly across Fabaceae species, ranging from a single gene in *Vicia faba* and *Pisum sativum* to 26 in *Glycine max* [32]. *A. thaliana* is another model species with extensively studied phloem-specific proteins. Initially, two SEO-related (SEOR) genes, *At3g01680* and *At3g01670*, were identified, encoding AtSEOR1 and AtSEOR2 proteins respectively [33], along with a third gene, *At1g67790*, which was initially considered a non-expressed pseudogene [32]. Recent studies employing single-cell RNA-sequencing techniques have revealed that this gene, encoding the AtSEORc protein, is actively transcribed in phloem cells [34]. Furthermore, the expression of all *Arabidopsis* SEOR genes has been specifically associated with the final stage of phloem differentiation [35].

Given the utility of P-proteins as markers for investigating long-distance molecular transport in plants, their study in tree model species is particularly intriguing. In *Malus domestica*, initially, only one SEOR encoding gene was identified [32]. However, research in another tree species, *Populus trichocarpa*, has shown the presence of NPB bodies in its sieve elements, which, unlike forisomes, do not respond to Ca^2+^ ions or cell wall wounding [36]. This remains the only study of SEOR proteins in *P. trichocarpa* thus far, revealing just one protein potentially orthologous to *Arabidopsis* AtSEOR1 [36]. Based on extensive genomic studies of *Populus* and other model plants, we investigated SEOR proteins in *P. trichocarpa*. Our genomic analysis uncovered 12 genes in the *P. trichocarpa* genome, potentially orthologous to three *A. thaliana* SEOR-encoding genes. Subsequent ultrastructural, transcriptomic, and proteomic analyses identified NPBs in *P. trichocarpa* sieve elements and delineated SEOR proteins with yet unknown function.

To standardize the nomenclature of phloem-specific proteins being used in this work, we define “P-protein” as a term for all phloem-specific proteins, defined historically and recently as elements of sieve element-specific protein bodies. SEO refers to proteins found in Fabaceae species that have been shown to have an occlusion function in sieve elements, while SEOR denotes proteins encoded by orthologues of SEO genes in other plant species, for which no occlusive function has been demonstrated so far.

## Materials and methods

### Plant material and growth conditions

**Fig. 1:**
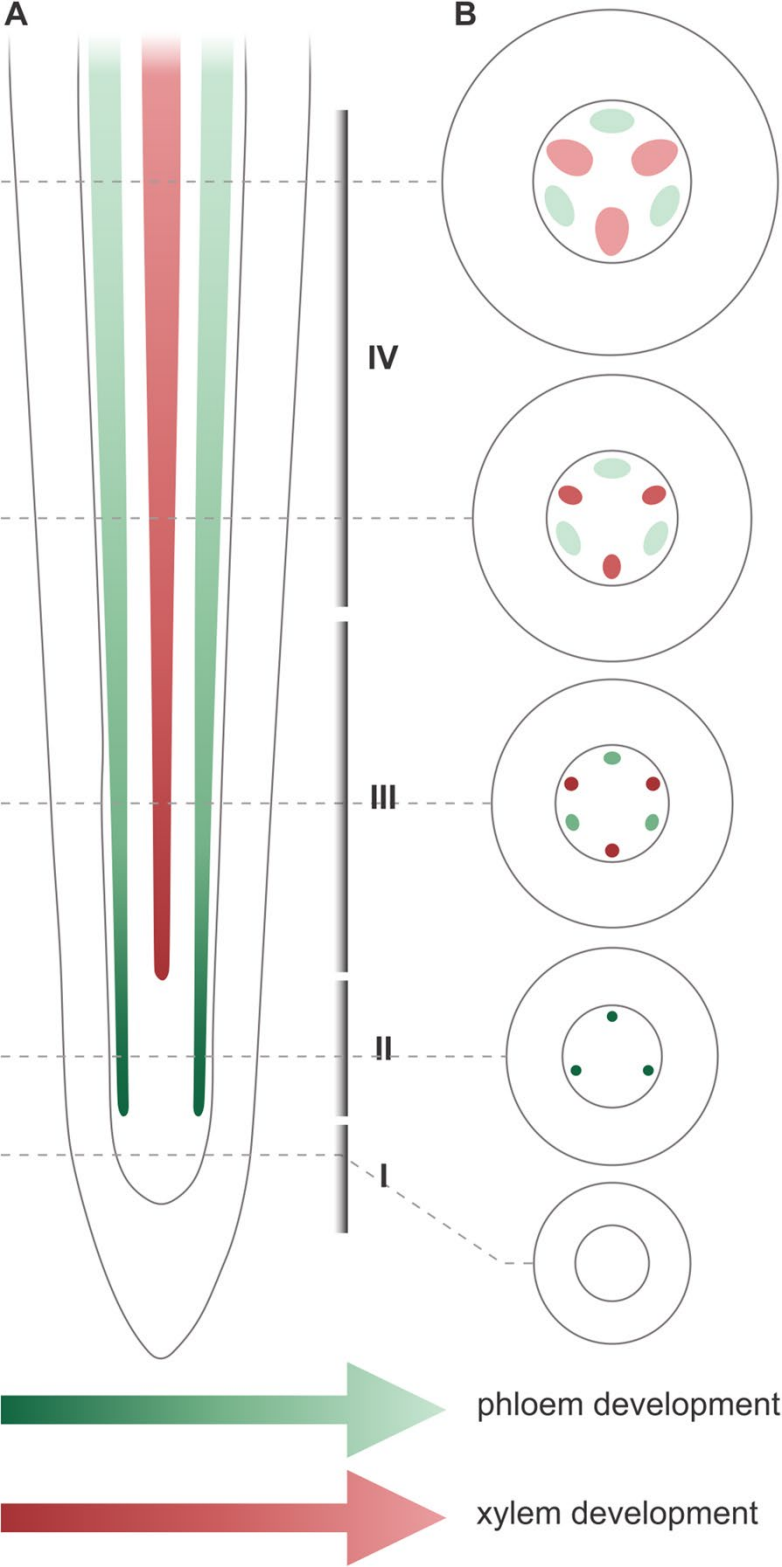
Illustrative overview of the primary vascular tissue developmental stages in the pioneer root of *P. trichocarpa*: A – tangenial section; B transverse sections. The phloem and xylem are represented in a gradient of green and red shades respectively, and not drawn to scale. I – meristematic tissue, II – phloem precursors, III – maturing phloem and xylem precursors, IV – mature phloem and maturing xylem

Experiments were conducted using black cottonwood (*Populus trichocarpa* Torr. & A. Gray ex Hook.) pioneer roots from plants germinated from seeds supplied by a seed bank. The initial cultivation phase occurred in a Conviron GR96 plant growth chamber, maintaining a 16/8-h day/night photoperiod at temperatures of 18/14 °C. Three-month-old plants were transferred to the experimental field site at the Institute of Dendrology, Polish Academy of Sciences in Kórnik, Poland (52°14′39.3′′N, 17°06′10.6′′E). Here, plants were grown in a semi-open foil tunnel greenhouse, with the plants positioned in rhizotrons arranged vertically within an underground chamber as previously described [37]. The pioneer roots were identified according to the methods outlined by Bagniewska-Zadworna et al. [38]. Fragments were subsequently sectioned corresponding to the primary vascular tissue developmental stages: I – meristematic tissue, II – phloem precursors, III – maturing phloem and xylem precursors, IV – mature phloem and maturing xylem (Fig. 1), according to Bagniewska-Zadworna et al. [38–40]. Additionally, stems (Sects. 0–2 cm from the apical meristem) and leaves (sections around 2–3 cm^2^ containing the midrib) were harvested for RT-qPCR analysis.

### Transmission electron microscopy (TEM)

Root fragments measuring 5 mm were immediately fixed in a freshly prepared solution containing 2% (v/v) glutaraldehyde and 2% (v/v) formaldehyde in 0.1 M cacodylic buffer (pH 7.3; Polysciences), followed by overnight incubation at 4 °C. Subsequent steps were adapted from methods optimized for phloem tissue [41], with minor modifications suitable for pioneer roots of *P. trichocarpa*. Samples were post-fixed in 1% osmium tetroxide in 0.1 M cacodylic buffer (pH 7.3; Polysciences) for 2 h, then counterstained with 2% uranyl acetate in 5% ethanol for 1 h in darkness at room temperature. Following dehydration in a graded ethanol series (10–100%, v/v), the samples were infiltrated with an ethanol series (3:1, 1:1, 1:3), culminating in 100% acetone, then embedded in Spurr's low-viscosity resin (Polysciences) through a series of acetone (1:1) mixtures and subsequently pure resin. Polymerization occurred at 60 °C for 72 h. Ultrathin sections were cut using an ultramicrotome EM UC6 (Leica-Reichert) with a diamond knife at a thickness of 0.1 μm, placed on formvar-coated copper grids, stained with 2% lead citrate and 2% uranyl acetate, and examined using an HT7700 transmission electron microscope (Hitachi, Tokyo, Japan) at 100 kV. At least five sections from different plants were analyzed.

### SEOR gene identification

Genes encoding SEOR proteins were identified through genome comparative analysis using BLAST (Basic Local Alignment Search Tool) with default parameters for protein–protein BLAST [42]. Peptide sequences from well-characterized *SEOR* genes in *A. thaliana* served as queries to search for potential orthologues in the *P. trichocarpa* genome version v4.1, available in Phytozome 13, The Plant Genomic Resource [43, 44].

### Genome distribution and conserved motif detection

Gene locations were mapped using the MAP tool to illustrate the distribution of identified *P. trichocarpa SEOR* genes across its genome. Conserved motifs among SEOR proteins were identified using the MEME tool (Multiple Em Motif Elicitation Version 5.5.5;) [45]. Detected motifs were further analyzed with SMART (Simple Modular Architecture Research Tool) [46] to determine if they correspond to any known protein domains listed in the Pfam database.

### Phylogenetic analysis

Phylogenetic trees were constructed for all identified genes from *P. trichocarpa*, their orthologues in *A. thaliana*, and other SEO/SEOR proteins known from several plant model species: *Medicago truncatula*, *Vicia faba*, *Pisum sativum*, *Cucumis sativus*, and *Malus* domestica. This analysis was based on encoded protein amino acid sequences using MEGA 11 (Molecular Evolutionary Genetics Analysis version 11) [47]. The maximum likelihood method was employed, utilizing the Jones-Taylor-Thornton (JTT) substitution model and the bootstrap method with 1000 replications to test phylogeny.

### Analysis of gene expression by single-nucleus RNA-sequencing

Single-nucleus RNA-sequencing (snRNA-Seq) was conducted on nuclei isolated from pioneer root fragments corresponding to the primary stages of vascular tissue development (Fig. 1, stages I-IV). The method for nuclei isolation was adapted from Dorrity et al. [48] with minor modifications for *Populus* roots. Isolated nuclei were stained with DAPI (SIGMA; excited with a 405 nm laser) and sorted using a BD FACSAria™ Fusion flow cytometer (Becton Dickinson, USA) equipped with a 100 µm nozzle. The sorting protocol involved removing debris and selecting single nuclei based on a positive DAPI signal. Post-sorting, nuclei were concentrated by centrifugation at 500 g for 5 min and resuspended in 1 × PBS containing 0.04% BSA. Nuclei quality was assessed using an ImageStream^X^ MkII imaging flow cytometer (Cytek Biosciences, USA; Additional file, Fig. S1).

Single-nucleus transcriptomic libraries were prepared with the Chromium Controller and the Chromium Next GEM Single Cell 3′ Reagent Kit (10 × Genomics, USA), following the manufacturer's instructions. Approximately 20,000 nuclei were combined with reverse transcription reagents and processed on the Chromium Next GEM Chip G to produce gel beads in emulsion (GEMs). This process released primers from the gel beads, facilitating the synthesis of barcoded cDNA from captured mRNA.

After incubation, GEMs were pooled, and the cDNA was purified using magnetic beads. The barcoded cDNA underwent PCR amplification, followed by enzymatic fragmentation and size selection. Illumina P5 and P7 adaptors, i7 and i5 sample indexes, and the TruSeq Read 2 sequencing primer site were added through end repair, A-tailing, adaptor ligation, and PCR, resulting in the completion of the final libraries. Sequencing was conducted on the NovaSeqX 10B platform (Illumina) by Macrogen Europe (Netherlands).

### snRNA-seq data analysis

Raw sequencing data were processed using Cell Ranger v7.1.0 (10 × Genomics). A reference genome for *P. trichocarpa* (black cottonwood; isolate Nisqually-1) was prepared using the '*cellranger mkref*' command, incorporating the *P. trichocarpa* v4.1 genome assembly and gene annotations from Phytozome v13 (BioProject PRJNA10772, Assembly GCA_000002775.4) [44, 49, 50]. The '*cellranger count*' command was executed with default settings using STAR v2.7.2a [51] as the aligner. The resulting gene-nucleus matrices were analyzed using the Seurat package v5.1.0 [52] in R v4.2.1. Low-quality nuclei were excluded based on the following criteria: gene counts > 200, UMIs < 20,000, mitochondrial content < 5%, and log10(gene count per UMI) > 0.8. Only genes detected in at least five nuclei were included. Samples were combined using the '*merge*' function, and each was normalized separately using the '*sctransform v2*' function [53] implemented in Seurat. Dimensionality reduction was conducted on 3,000 highly variable features. The '*FindNeighbors*' function was applied with 51 principal components for the full datasets, and clustering was performed using the Louvain algorithm. Nucleus clusters were visualized with UMAP and annotated based on the expression of known marker genes. The '*NormalizeData*' function was used for differential gene expression analysis and plotting within the Seurat RNA assay. Gene markers for each nucleus cluster were identified using the '*FindMarkers*' function, with differentially expressed genes defined by an adjusted p-value ≤ 0.05, pct.1 > 0.5, and pct.2 < 0.3.

### Analysis of gene expression by RT-qPCR

RNA isolation was conducted using the Ribospin™ Plant kit (GeneAll Biotechnology Co., Ltd, Seoul, South Korea) as per the manufacturer’s protocol. cDNA synthesis followed, utilizing the High-Capacity cDNA Reverse Transcription kit (Applied Biosystems, Thermo Fisher Scientific Inc., Waltham, MA, USA). A 1:10 dilution of cDNA was used for the PCR reactions. The RT-qPCR (Reverse Transcriptase quantitative Polymerase Chain Reaction) was performed with the SYBR Green Master Mix kit (Applied Biosystems) in a CFX96 Touch Real-Time PCR Detection System (Bio-Rad, Hercules, CA, USA). The PCR program included an initial denaturation by hot start at 95 °C for 10 min, followed by 40 cycles of a three-step thermal cycling protocol: denaturation at 95 °C for 15 s, annealing at 60 °C for 1 min, and extension at 72 °C for 15 s. *Actin* and *UBQ* (ubiquitin) were selected as housekeeping genes due to their consistent expression across all cells and time points. Multiple primer pairs, specific to one or more SEOR encoding genes, were employed (Tab. 1), as some genes are highly similar, and making gene-specific primer design was impossible. Data analysis was conducted using the 2^−ΔΔCt^ method [54]. Each analysis included three technical replicates for each of the three biological replicates across four experimental variants. Statistical analyses were conducted using Statistica 10 software (StatSoft Poland Inc., Tulsa, OH, USA) (Additional file, Fig. S2).

**Table 1:** Primers used in RT-qPCR analysis

Name	Gene	Primer F 5′ ➝ 3’	Primer R 5′ ➝ 3’
PP1	*Potri.017G071000* *Potri.001G340600* *Potri.001G340500* *Potri.001G340400* *Potri.001G340300* *Potri.001G340200*	AGGAGTGTTCTGGCTTGTG	TCATCCTTGGCGTAAATCAGG
PP2	*Potri.012G090400*	TGGTAGCACAATGATGAAGGC	TCATATGTACCCTCTACGATGGC
PP3	*Potri.008G183200*	TGCAAAAGCAGTTGCGAAAG	AGGTTCTCGTTTACAATAACACCG
PP4	*Potri.010G050300*	CTCCTGATGGCCGTGAATT	CATCAAGTTGTGCTTGATGAGC
PP5	*Potri.010G050200* *Potri.010G050701*	CTCAGCTGTTGTGAACTACACA	TCGAAGTTGAGAAGGGGTATG
PP6	*Potri.010G050501*	AACTCCATACCCCATTCCAC	TTACATGATGAACTTCTCCAGGG
Actin	*Potri.001G309500*	GCCCAGAAGTCCTCTTCCAG	AAGGGCGGTGATCTCCTTG
Ubiquitin	*Potri.005G096700*	AGGAACGCGTTGAGGAGAAG	TATAAGCAAAAACCGCCCCTG

### Proteomic analysis

Proteomic analysis utilized pioneer root fragments corresponding to four vascular developmental stages [40]. Three biological replicates were processed for protein isolation from 250–350 mg of fresh root fragments, each comprising a pool of roots from 3–5 plants, using a modified phenol extraction method [55].

Plant material was pulverized in liquid nitrogen and extracted in extraction buffer (0.7 M sucrose; 0.5 M Tris; 50 mM EDTA; 0.1 M KCl; 2% β-mercaptoethanol; 2% DTT; 2 mM PMSF; 1% insoluble PVP; pH 7.5; 1 ml per 200 mg of weight) for 15 min on ice. Water-saturated phenol with 0.1% 8-hydroxyquinoline (to achieve a yellow coloration) was added, and the mixture was shaken continuously for 10 min. Following centrifugation, the phenol phase was collected and mixed 1:1 with extraction buffer, shaken for 5 min, then centrifuged again. The phenol phase was then mixed with five volumes of ice-cold 0.1 M ammonium acetate in methanol and precipitated at −20 °C for 2 days. Proteins were pelleted by centrifugation, washed twice with ammonium acetate solution and acetone, then air-dried. Proteins were resuspended in rehydration buffer (100 mM ammonium bicarbonate with 0.5% SDC in water), and concentrations were estimated using the Pierce BCA Protein Assay Kit (ThermoFisher Scientific), according to the manufacturer’s protocol. For LC–MS/MS sample preparation, 20 µg of protein per sample was dissolved in 25 µl of 50 mM ammonium bicarbonate.

LC–MS/MS sample preparation included protein digestion with trypsin. Samples were initially incubated with DTT for 5 min at 95 °C, followed by alkylation with IAA for 20 min in darkness at room temperature. Samples were then digested with trypsin (2 µl) for 18 h at 37 °C. After digestion, 1.5 µl of 10% TFA was added, and samples were vortexed for 10 min, centrifuged, and the peptide-containing supernatant was transferred to new vials for LC–MS/MS analysis performed by Laboratory of Mass Spectrometry in Institute of Bioorganic Chemistry, Polish Academy of Sciences.

Total spectrum count was employed to quantify protein levels in the tested root fragments corresponding to various phloem developmental stages (I-IV). Statistical analyses were performed using Statistica 10 software (StatSoft Poland Inc., Tulsa, OH, USA).

## Results

### Detection of P-protein bodies in *Populus trichocarpa* sieve elements in transmission electron microscopy analyses

**Fig. 2:**
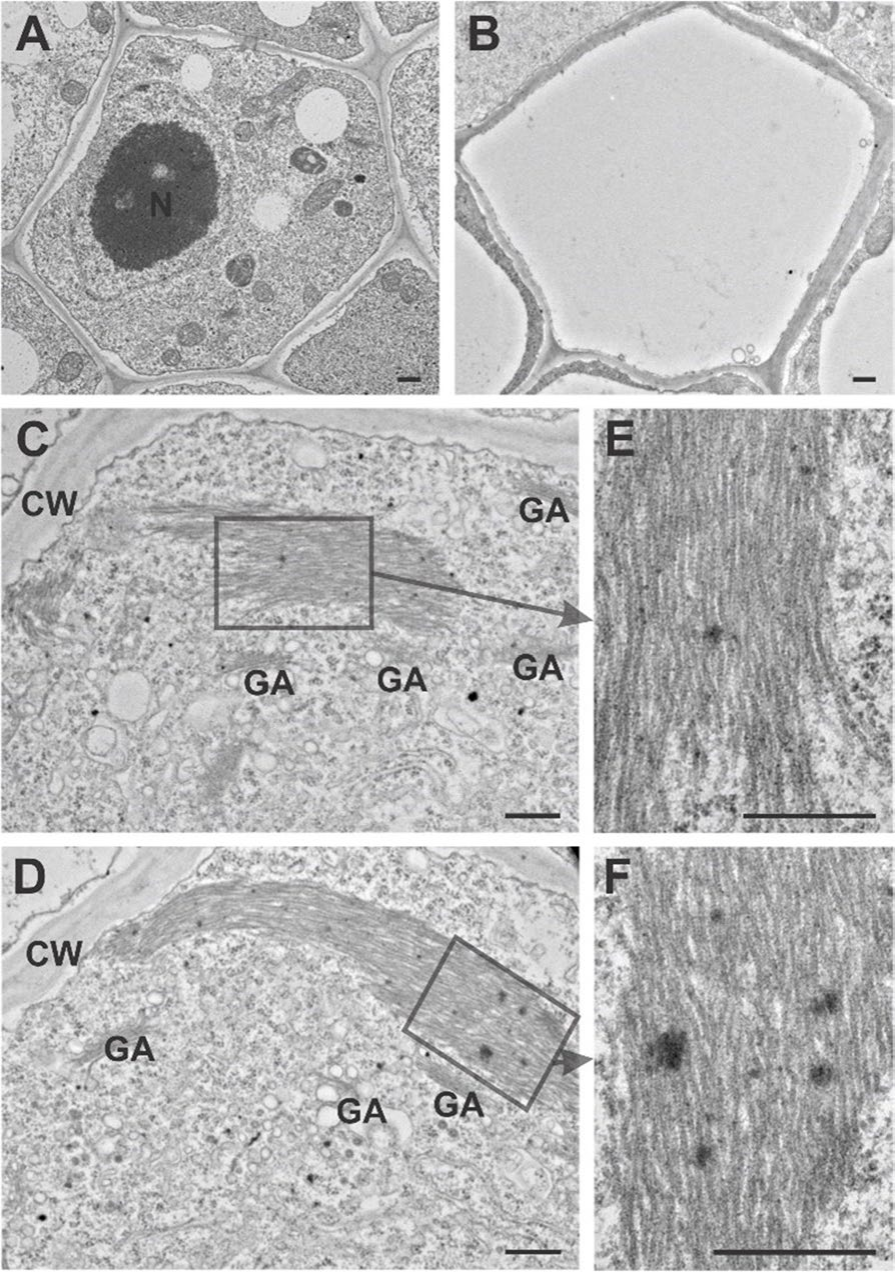
Sieve elements and P-proteins in the pioneer root of *P. trichocarpa*. An undifferentiated sieve element with electron-dense cytoplasm containing all organelles (corresponds to stadium II in Fig. 1). B Mature sieve element with electron-translucent cytoplasm, devoid of the nucleus and most other organelles (corresponds to stadium IV in Fig. 1). **C**-**D** P-proteins in different cross sections of the same sieve element (corresponds to stadium III in Fig. 1). **E**–**F** Regularly arranged bundles of P-protein filaments (corresponds to stadium III in Fig. 1). N – Nucleus, CW – Cell Wall, GA – Golgi Apparatus, scale bars = 500 nm

Sieve elements in the pioneer roots of *P. trichocarpa* were distinguished from other phloem cell types by their regular, mostly pentagonal shape in cross-section (Fig. 2A, 2B). Unlike both parenchyma and companion cells, differentiating SEs lack a large central vacuole (Fig. 2A). The defining feature of phloem differentiation is the reduction of cytoplasm in conducting cells, which lack nuclei and most other organelles (Fig. 2B). Only mitochondria, plastids, smooth endoplasmic reticulum, and small vesicles persisted in mature SEs, all of which were reduced in number and anchored to the cell wall. However, the most definitive marker of SEs was the presence of P-proteins, already visible in cells with dense cytoplasm that were still developing. P-proteins form filamentous structures in the sieve element lumen, closely associated with the cell wall (Fig. 2C-D). These filaments, with a diameter of ~ 24 nm, were primarily aligned in parallel bundles (Fig. 2E-F). Various cross-sections revealed that filaments may change direction, indicating that the arrangement of the entire P-protein bundle is more complex than initially perceived.

### *SEOR* gene family in *Populus trichocarpa*

**Fig. 3:**
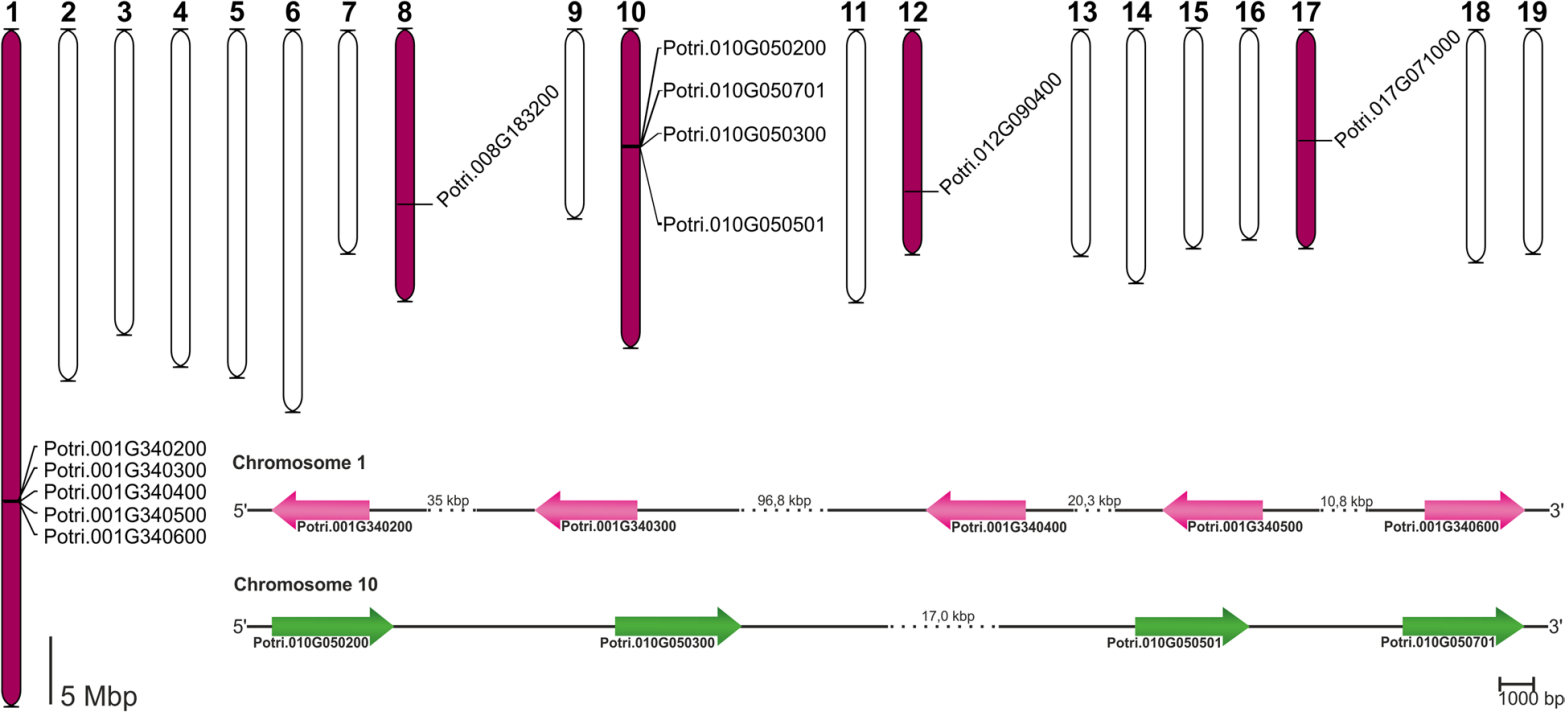
Chromosomes of *P. trichocarpa* showing the locations of all identified SEOR encoding genes. Chromosomes harboring at least one *SEOR* gene are colored purple. Additionally, position of SEOR-encoding genes identified on chromosome 1 (pink) and 10 (green)

Analysis of the latest version of the *P. trichocarpa* genome (v4.1) aimed to identify all potential orthologues of known *A. thaliana* SEOR genes, encoded by *At3g01680*, *At3g01670*, and *At1g67790*. BLASTp searches, based on sequence homology, identified 12 genes potentially encoding SEOR proteins in *P. trichocarpa* (Fig. 3). Selection of *P. trichocarpa SEOR* orthologues was based on an E-value threshold of less than 1e^−30^ to mitigate false positives or negatives [56] and a sequence identity above 70%. The peptides encoded by the selected genes were all 684–723 amino acids in length.

To explore the evolutionary trajectory of *SEOR protein* genes in *P. trichocarpa*, their genomic locations were analyzed. All identified *SEOR protein* genes were exclusively found on chromosomes 1, 8, 10, 12, and 17, with none located on the other 14 chromosomes (Fig. 3). Chromosomes 1 and 10 hosted multiple *SEOR protein* genes – five and four genes, respectively (Fig. 3). Three genes were individually located on different chromosomes (Fig. 3).

**Fig. 4:**
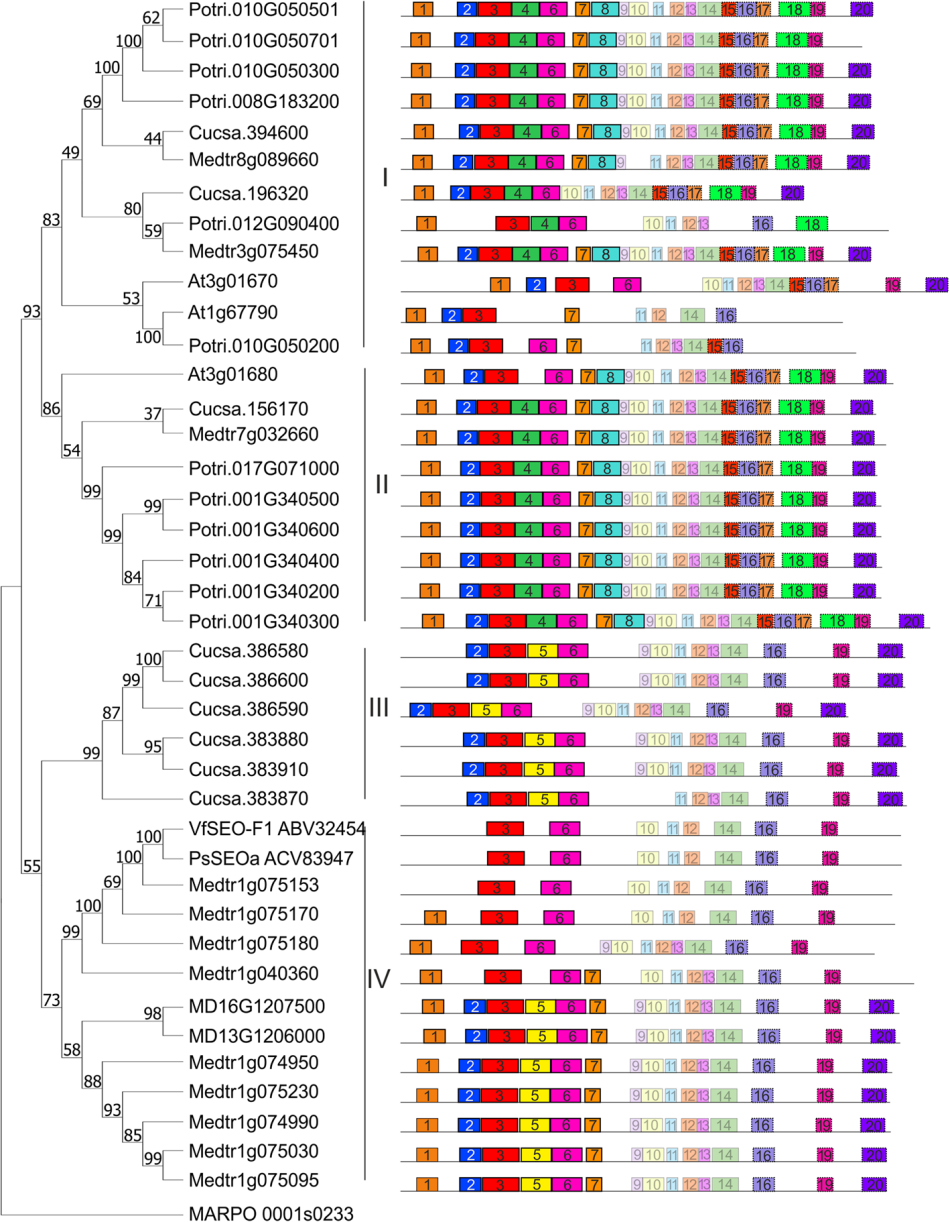
Phylogenetic tree of SEO/SEOR genes from model species: *P. trichocarpa* (Potri), *A. thaliana* (At), *Medicago truncatula* (Medtr), *Vicia faba* (Vf), *Pisum sativum* (Ps), *Cucumis sativus* (Cucsa), and *Malus domestica* (MD) with identified conserved peptide motifs. Peptide motifs identified as fragments of SEO_N and SEO_C domains are fully colored and framed with a solid and dashed black line, respectively. Unidentified motifs are presented as partially transparent

Phylogenetic analysis using a maximum likelihood approach produced an unrooted tree, modeling the evolutionary relationships of SEO/SEOR proteins between *P. trichocarpa* and several other model species (Fig. 4). *Populus* proteins were categorized into two groups, labeled I and II, containing 6 proteins each (Fig. 4). Group I contains proteins encoded by genes located on chromosome 8, 10 and 12 (Fig. 4). Group II consists poplar proteins encoded by genes located on chromosome 1 and 17 (Fig. 4). Poplar SEOR proteins are grouped with all *Arabidopsis* SEOR proteins and single SEO/SEOR from other model species (two *A. thaliana,* two *C. sativus* and two *M. truncatula* SEO/SEOR proteins in group I; one *A. thaliana,* one *C. sativus* and one *M. truncatula* SEO/SEOR proteins in group II). Most SEO proteins in Fabaceae species are grouped separately in group III and IV with two from *M. domestica* (Fig. 4). Additionally, an analysis of conserved peptide motifs using the MEME online tool (http://meme.nbcr.net/) [57] identified 20 motifs (Fig. 4), detailed in Additional file (Table S1). All motifs found were annotated using SMART (Simple Modular Architecture Research Tool) [46, 58] and the Pfam database [59], with 14 identified as fragments of the SEO_N (motifs 1–8) and SEO_C (motifs 15–20) domains, characteristic for SEO/SEOR proteins.

### *SEOR* gene expression

**Fig. 5:**
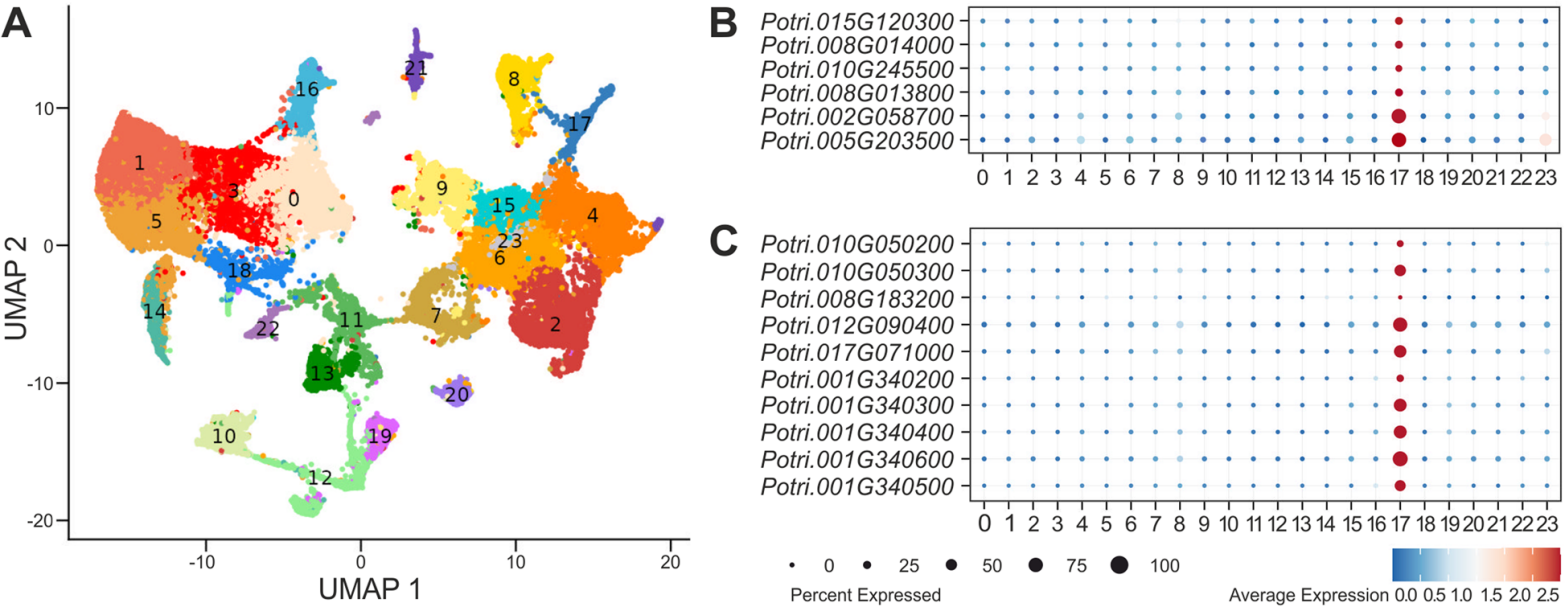
**A** UMAP visualization of 24 cell clusters in the *P. trichocarpa* root. Dots represent single nuclei with colors denoting clusters. **B** Expression pattern of known sieve element marker genes. **C** Expression pattern of identified *SEOR* genes across all clusters. Dot diameter indicates the proportion of cluster nuclei expressing the gene, and color denotes the average relative expression of a particular gene in each cluster

Analyses of SEOR encoding gene expression were conducted using single-nucleus RNA-sequencing (snRNA-seq), which has been recently adapted to plant research [60], and additionally RT-qPCR technique. snRNA-seq analysis targeted whole pioneer roots, encompassing both primary phloem and xylem, whereas RT-qPCR focused on root fragments corresponding to identified vascular tissue development stages (I-IV). Additionally, organ specificity of selected gene expression was assessed using RT-qPCR on leaf and stem fragments. The pre-processing of snRNA-seq data facilitated the detection and removal of low-quality nuclei. Consequently, over 33,000 root nuclei from two highly correlated biological replicates were profiled, demonstrating the high quality of the data. Subsequently, nuclei were clustered into 24 groups based on their expression profiles and visualized in 2D Uniform Manifold Approximation and Projection (UMAP) space (Fig. 5A). Among these clusters, cluster 17, containing 600 nuclei, was identified using six known marker genes [61, 62] (Fig. 5B) as representing phloem sieve elements (Fig. 5). Notably, 589 of these nuclei (over 98%) expressed at least one *SEOR* gene. The snRNA-seq analysis revealed the expression of 10 out of the 12 identified SEOR encoding genes, all of which were expressed: *Potri.010G050200*, *Potri.010G050300*, *Potri.008G183200*, *Potri.010G090400*, *Potri.017G071000*, *Potri.001G340200*, *Potri.001G340300*, *Potri.001G340400*, *Potri.001G340500*, *Potri.001G340600* (Fig. 5C). All these genes were specifically expressed in nuclei from cluster 17 (Fig. 5C). Additionally, *SEOR* gene expression was significantly higher in nuclei corresponding to sieve elements (Fig. 5C). However, the percentage of nuclei exhibiting high average expression of *SEOR* genes (over 1.5) varied among individual genes, from less than 1% for one gene (*Potri.008G183200*) to between 20% and nearly 80% for others (*Potri.010G340600*) (Fig. 5C; Available data).

**Fig. 6:**
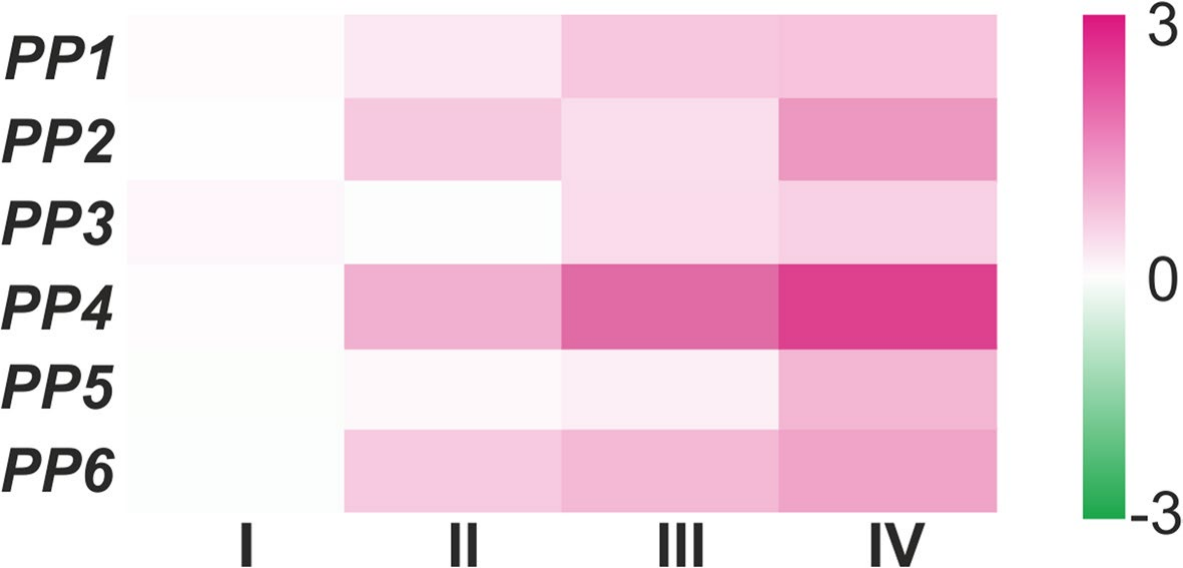
Heatmap visualization of relative expression of *P. trichocarpa SEOR* genes in four root developmental stages (I-IV) using six pairs of primers specific for one or overlapping sequences of 2/6 genes, as detailed in Tab. 1. Relative quantification for each primer pair-reaction was normalized according to actin and ubiquitin gene expression. Pink indicates up-regulation relative to stage I, which represents the apical meristem (with no differentiating phloem cells), and green indicates down-regulation. Color intensity represents expression levels

Parallel to snRNA-seq, RT-qPCR analysis was performed on root fragments representing subsequent vascular tissue developmental stages, ranging from meristematic tissue (stage I), through phloem precursors (stage II), phloem maturation (stage III), to mature phloem with developing xylem (stage IV). Due to the high similarity and sequence overlap among the studied genes, it was not feasible to design a specific pair of primers for each *SEOR* gene. Consequently, some primer pairs were designed to overlap several gene sequences (Tab. 1). Almost all tested primer pairs exhibited similar expression profiles, with relative expression levels increasing across the developmental stages (Fig. 6A). The highest gene expression for each was observed at stage IV, characterized by fully differentiated phloem cells (Fig. 6A). Additionally, gene expression was evaluated in stem and leaf tissues, and reaction with every primer pair gave the same PCR-product as in root samples (data not shown).

### SEOR protein identification in LC–MS/MS analysis

**Fig. 7:**
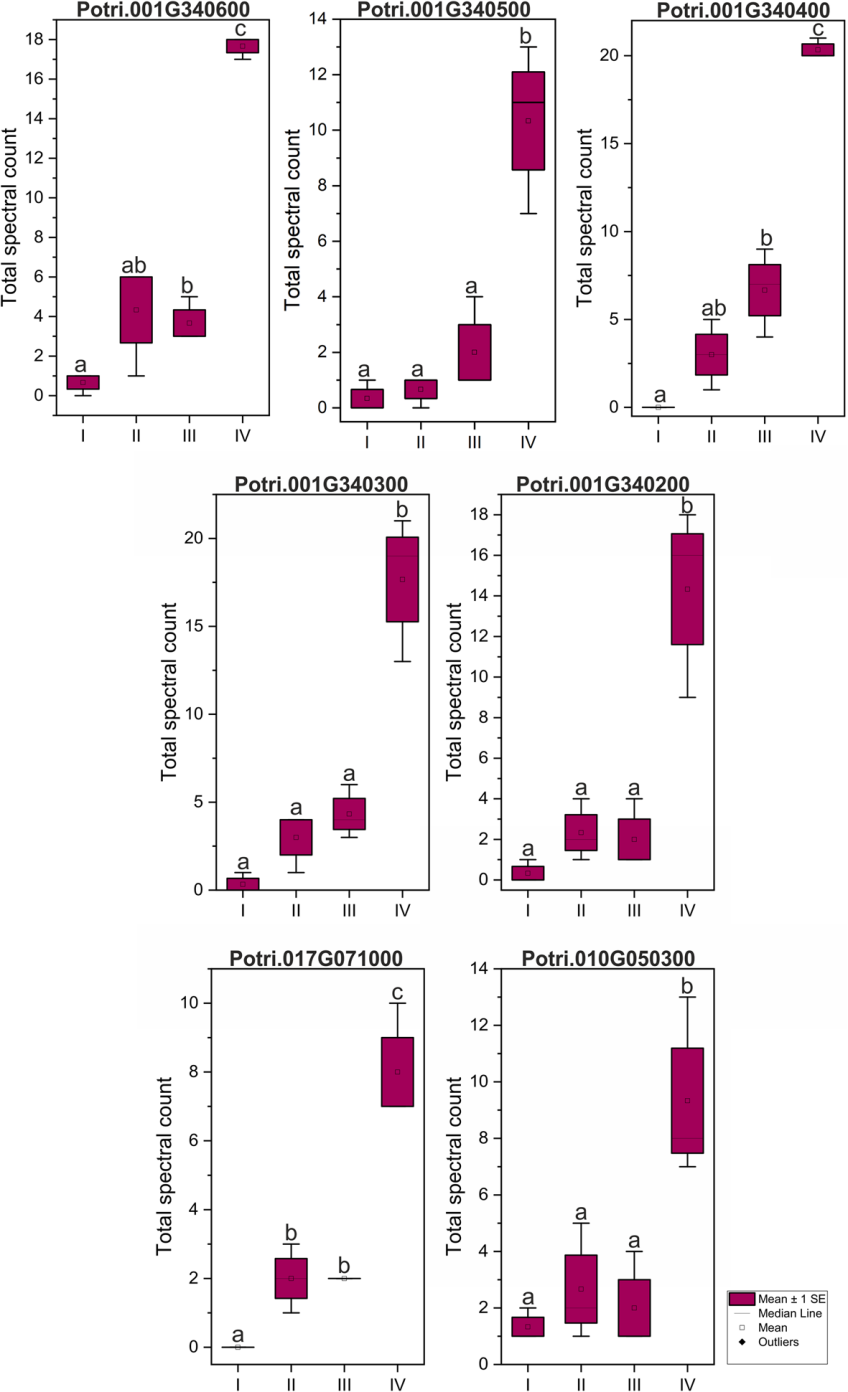
Normalized total spectral counts for identified SEOR proteins across root developmental stages I-IV, from three biological replicates, showing mean values ± standard error. Statistical analysis: one-way ANOVA with Tukey post-hoc test, *p*-value < 0.05

Proteome analysis using LC–MS/MS techniques confirmed the presence of seven SEOR proteins in *P. trichocarpa* root samples (Fig. 7). All of them were also detected at the transcriptomic level via snRNA-seq (*Potri.001G340600*, *Potri.001G340500*, *Potri.001G340400*, *Potri.001G340300*, *Potri.001G340200*, *Potri.017G071000*, *Potri.010G050300*). Additionally, the expression analyses at the transcriptomic and proteomic levels showed divergence for three proteins, which gene expression was observed without detectable protein levels (*Potri.008G183200*, *Potri.010G050200*, and *Potri.012G090400*) (Figs. 5, 7). Moreover, the proteome analysis facilitated quantitative assessments. An increasing trend in the abundance of identified SEOR proteins was noted through the root developmental stages (I-IV) (Fig. 7). Notably, at stage IV, which features fully developed phloem cells, the quantities of all identified P-proteins, as measured by total spectrum count, were significantly higher (Fig. 7).

## Discussion

Mature sieve elements are almost devoid of cytoplasm, yet they contain characteristic protein structures whose functions are mostly unknown or unclear. However, the cellular specificity of these structures suggests a significant role in sieve element functionality.

In our study, we observed phloem-characteristic protein agglomerates in developing sieve elements using transmission electron microscopy (TEM) (Fig. 2). These structures are reminiscent of those observed in maturing *Nicotiana tabacum* sieve elements [6, 63]. In tobacco, phloem protein imaging across immature and mature sieve elements typically shows one agglomerate per cell, occasionally positioned near the plasma membrane or more frequently on sieve plates [64]. It has been hypothesized that these phloem-specific proteins contribute to sieve plate occlusion by forming a plug upon cellular wounding, though this function remains controversial [64–66].

Historically, SEO, being family of phloem-specific proteins, were identified exclusively within the Fabaceae species as structural elements of forisomes. Subsequent discoveries of SEO encoding gene orthologues in numerous non-Fabaceae plants, which lack forisomes, have led to ambiguities regarding the general function of these proteins, thus, these genes have been collectively termed SEOR. To date, only two specialized types of P-proteins have been characterized: the Fabaceae-specific SEO proteins being part of forisomes and the Cucurbitaceae-specific PP1. Forisomes are capable of undergoing conformational changes induced by calcium ions, which facilitate the opening or plugging of sieve elements following wounding and promote subsequent plant regeneration [29, 67]. This functionality has been validated through immunological studies in the model legume species *M. truncatula*, which possesses three SEO proteins [27, 68]. The other functionally characterized P-protein, PP1, identified in *Cucurbita maxima*, forms filamentous structures within the slime plugs of sieve elements [69]. Intriguingly, the *C. maxima* PP1 protein lacks significant sequence similarity or specific domains associated with SEO proteins, suggesting it should not be categorized within the SEO gene family. Nonetheless, it may perform a similar, unique function in Cucurbitaceae plants, as no *PP1* orthologues have been found in other species [32, 70]. Despite the characterization of these two types of P-proteins, the molecular nature and function of phloem proteins, found across many mono- and dicotyledonous species, remain elusive. Notably, the number of SEO/SEOR encoding genes varies significantly among plant species, ranging from a single gene in species such as *Pisum sativum* and *Canavalia gladiata* to 55 genes in *Cercis canadensis* (Tab. 2; based on [32] and Phytozome database). To bridge the knowledge gap concerning P-proteins, we conducted a comprehensive analysis in the tree model species *P. trichocarpa*. Our multidisciplinary research spanning genomic, transcriptomic, and proteomic levels concludes that fully developed sieve elements, though largely devoid of cytoplasm, still harbor specific protein structures.

**Table 2:** The number of identified SEO and SEOR genes mentioned by Rüping [32] and found in Phytozome database in the year of writing (in brackets)

Species	*SEO/SEOR* genes
*Medicago truncatula*	9 (13)
*Glycine max*	26
*Vicia faba*	1 (13)
*Canavalia gladiata*	1
*Pisum sativum*	1
*Malus domestica*	2 (16)
*Arabidopsis thaliana*	3
*Vitis vinifera*	15
*Solanum phureja*	3
*Amaranthus hypochondriacus*	6
*Anacardium occidentale*	12
*Arachis hypogaea*	38
*Camelina sativa*	9
*Brassica rapa*	5
*Carya illinoinensis* cv. ‘*Elliott*’	24
*Cercis canadensis*	55
*Corymbia citriodora*	46
*Fragaria* × *ananassa* cv. *‘*Royal Royce’	35
*Lotus japonicus*	11
*Solanum lycopersicum*	6
*Theobroma cacao*	8

Previously, only one *P. trichocarpa* gene was identified as a potential orthologue of the *A. thaliana* SEOR gene, *Potri.017G071000* [36]*.* Our comprehensive genome-wide analysis has revealed that the *P. trichocarpa* genome contains 12 SEOR encoding genes (Fig. 3), representing the highest number identified in any tree model species to date.

The organization of SEO/SEOR encoding genes within genomes is intriguing aspect of this gene family. Previous studies have shown that many SEO genes are arranged in tandem duplications across various model species. In *M. truncatula*, this applies to seven out of nine genes on chromosome 1, *V. vinifera* to three and eight genes on chromosomes 1 and 14, respectively, and in *G. max* to five and six genes on chromosomes 10 and 20, respectively [32]. Our analysis also identified SEOR genes being arranged in tandem duplications in *P. trichocarpa* on chromosomes 1 and 10 containing five and four SEOR encoding genes respectively. This pattern suggests that the tandem arrangement of SEO/SEOR-protein encoding genes might be a common phenomenon among plant species. The organization of these genes reflects the evolutionary history of the *Populus* genome, shaped by duplication events. The most recent salicoid-specific duplication impacted around 92% of the genome [50]. It was followed by a series of rearrangements, featuring tandem fusions and duplications, also local, potentially including segments containing SEOR encoding genes.

Originally, P-proteins were believed to form only immobilized structures within individual sieve elements [71, 72]. However, subsequent research demonstrated that P-proteins could undergo long-distance transport across graft unions [73]. Intriguingly, while the trafficking involves proteins, SEOR-protein mRNA was not detectable in the tested *Cucumis sativus* scion [73], contrasting with reports of mRNA transport from companion cells to sieve elements [74]. In *Cucurbita* species, SEOR-protein mRNA has been found to accumulate exclusively in companion cells [70, 75]. Our snRNA-seq analysis did not reveal extensive expression of SEOR encoding genes in companion cells, which were identified as cluster number 8 (Fig. 5).

One hypothesized function of SEO/SEOR-proteins is to protect the phloem or respond to phloem-feeding insects and fungal pathogens. To date, this function remains unconfirmed. However, it has been shown that SEOR-proteins can specifically interact with OGA^488^ [76], an oligosaccharide used for chitin detection. Chitin is a component of phloem-feeding insect stylets and fungal pathogen cell walls. The biological significance of SEOR-protein binding to OGA^488^, whether incidental or functional, remains unclear. Additionally, non-dispersive phloem-protein bodies in *P. trichocarpa*, composed of SEOR proteins, do not respond to cellular wounding [36], which does not support the proposed protective function.

Transcriptomic investigation on *P. trichocarpa* stem has shown that seven SEOR-encoding genes may be expressed: Potri.001G340200, Potri.001G340300, Potri.001G340400, Potri.001G340600, Potri.010G050300, Potri.012G090400, Potri.017G071000 [61]. Our single-nucleus RNA sequencing analysis revealed 10 expressed SEOR genes, what may indicate differences in the SEOR-gene expression between poplar organs.

Proteomic investigations of sieve elements are challenging due to difficulties in isolating them; most studies have utilized phloem sap, which varies in protein composition by plant organ, age, or experimental conditions [77]. Phloem sap contains only the soluble proteome and lacks immobile proteins essential for sieve element functions [78]. To date, SEOR proteins have not been observed in phloem sap, with the sole exception of a potential orthologue of *Arabidopsis* SEOR1 (*At3g01680* encoded) found in the exudate from *C. maxima* [28]. Our proteomic research, which identified seven SEOR proteins, suggests that P-protein bodies in *P. trichocarpa* may comprise more than the single protein previously reported [36].

Our findings indicate that both gene expression and protein levels are significantly elevated in the fourth developmental stage of the root, correlating with pseudotime data from single-cell RNA sequencing of *Arabidopsis* root, which shows all SEOR encoding genes being highly expressed at the final stage of phloem development [35]. SEO/SEOR encoding genes are prevalent throughout the plant kingdom, having been identified in numerous mono- and dicotyledonous plant species.

Our investigation into the *SEOR* genes in *P. trichocarpa* marks the first examination of this surprisingly large gene family, likely formed through numerous duplication events within the *Populus* genome. These proteins are hypothesized to contribute to the specific protein structures observed in sieve elements (Fig. 2). However, the precise functions of these SE-specific proteins remain elusive. Given their prevalence in most studied plant species, uncovering their role in phloem cells could be pivotal. Potential functions may include the modulation of pore opening/closing between neighboring sieve elements or providing structural support to sieve elements, which are nearly devoid of cellular content. Another potential role for SEOR-proteins could be related to phloem-dependent systemic signaling, as the phloem is a critical component in the long-distance transport of numerous physiologically significant molecules, such as photoassimilates [79, 80] and signaling molecules (proteins, mRNA, hormones, and lipids) [81–83]. This function is particularly vital for plant development under both physiological conditions and in response to biotic and abiotic stresses.

In this study, ultrastructural observations of maturing phloem cells were integrated with genomic, transcriptomic, and proteomic approaches to conduct a comprehensive identification and analysis of SEOR proteins in the model tree species, *P. trichocarpa*. This multi-omic approach has enabled us to characterize proteins that are likely crucial for sieve element function. The certain function of these proteins is still unknown despite over a century of research, however there are some reports indicating that SEOR function may be connected to the defense response to biotic stresses. Ultimately, our study lays the groundwork for future functional studies of phloem cells, which are technically challenging but increasingly feasible. Advancing our understanding of phloem physiology is of paramount importance, especially in the context of ongoing climate change.

## Supplementary Information

Supplementary Material 1.

## Data Availability

The datasets generated and analysed during the current study are available in the RepOD repository, [https://repod.icm.edu.pl/privateurl.xhtml?token=e9dd8cc5-9051-48f1-a813-ca3f7e202ddb] and GEO database [https://eur01.safelinks.protection.outlook.com/?url=https%3A%2F%2Fwww.ncbi.nlm.nih.gov%2Fgeo%2Fquery%2Facc.cgi%3Facc%3DGSE281380&data=05%7C02%7Ckarolina.kulak%40amu.edu.pl%7Ccf0435b38c3e42a0a2e408dd03bb9160%7C73689ee1b42f4e25a5f666d1f29bc092%7C0%7C0%7C638670826323633465%7CUnknown%7CTWFpbGZsb3d8eyJFbXB0eU1hcGkiOnRydWUsIlYiOiIwLjAuMDAwMCIsIlAiOiJXaW4zMiIsIkFOIjoiTWFpbCIsIldUIjoyfQ%3D%3D%7C0%7C%7C%7C&sdata=lh%2FBy44VuSTzaOjckguoggtTNIUWyZ83vS6yplvagME%3D&reserved=0].

## Abbreviations

NPB: Non-dispersive P-protein body
RT-qPCR: Reverse transcriptase quantitative polymerase chain reaction
SE: Sieve element
SEO: Sieve element occlusion
SEOR: Sieve element occlusion-related
snRNA-seq: Single-nucleus RNA-sequencing
