# Supplementary material for: Identification of phloem-specific proteinaSEOus structure heterogeneity in sieve element of Populus trichocarpa
Source: BMC Plant Biol. 2025 Apr 10;25:456. doi: 10.1186/s12870-025-06439-4 (PMC11983751; doi:10.1186/s12870-025-06439-4)
Supplement: Supplementary file 1 — Supplementary Material 1. [file 12870_2025_6439_MOESM1_ESM.pdf]

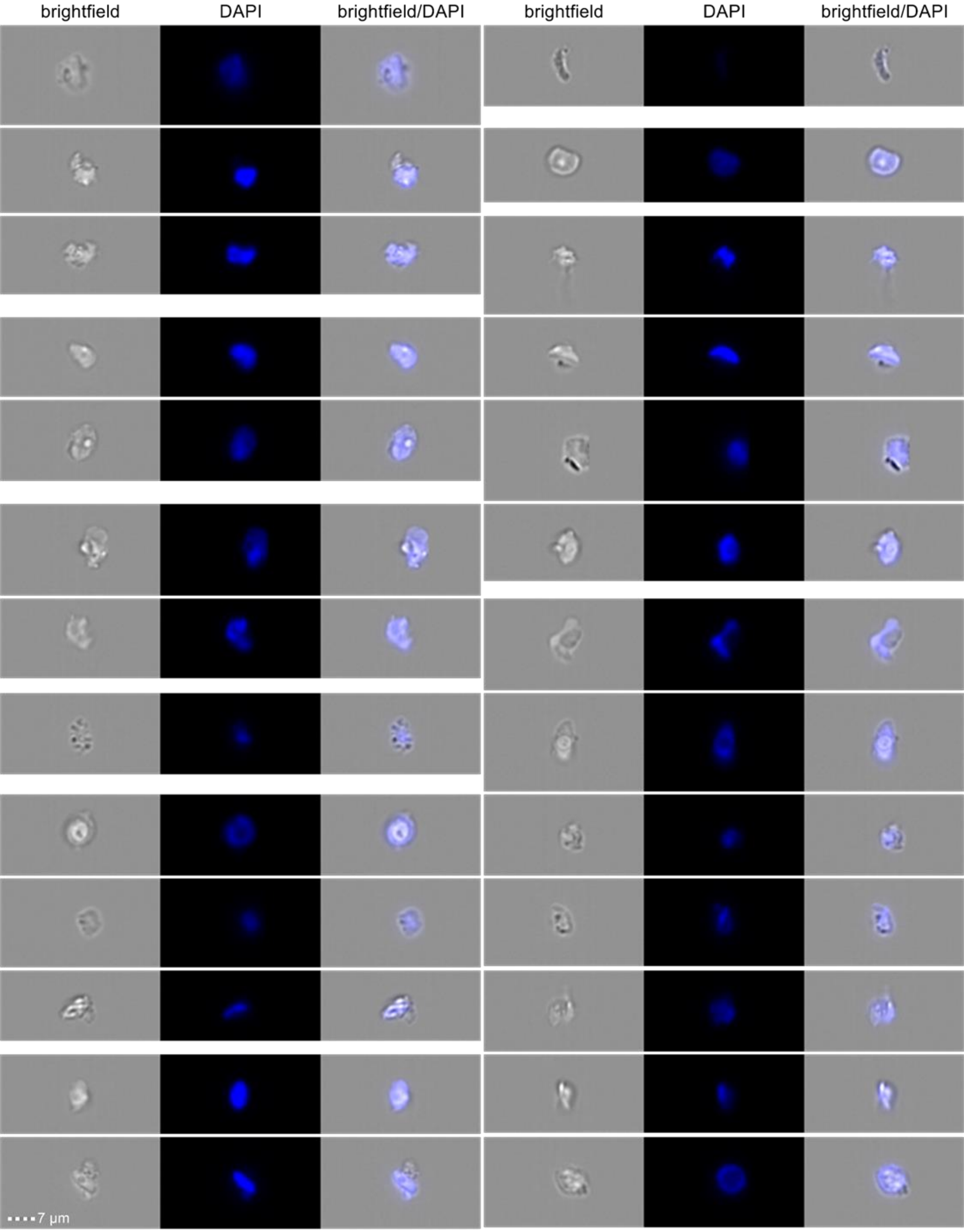

Figure S1. Nuclei quality check assessed using an ImageStream^{X} MkII imaging flow cytometer

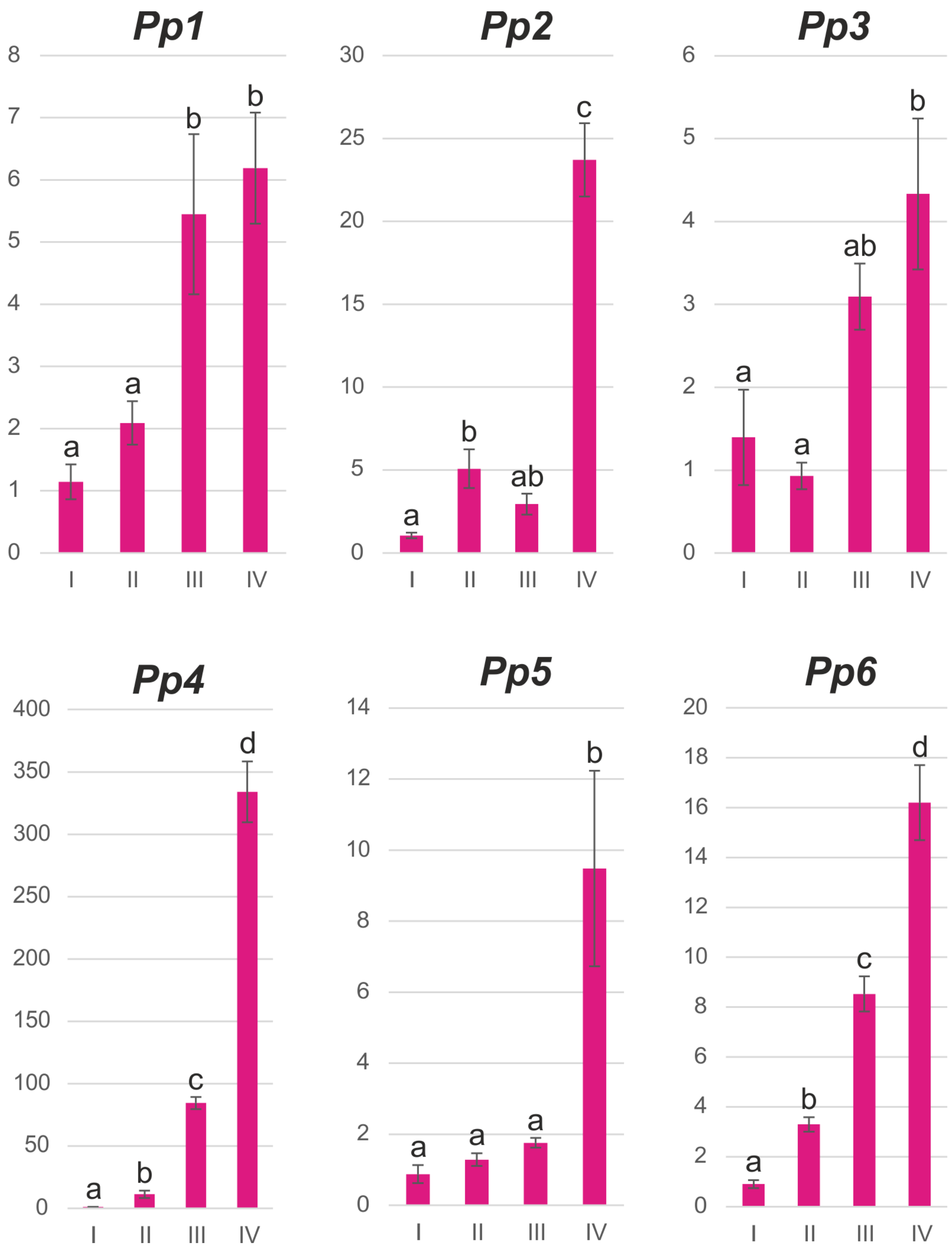

Figure S2. *P-protein* relative gene expression obtained for four root fragments with statistical analysis

Table S1. Sequences of conserved peptide motifs identified using MEME online tool

Motif number	Sequence of identified motif
1	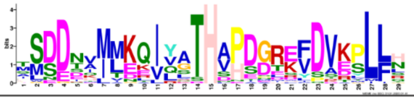
2	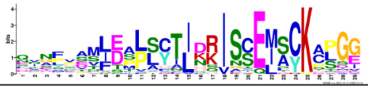
3	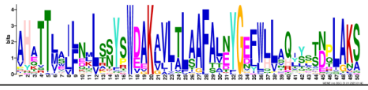
4	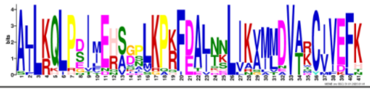
5	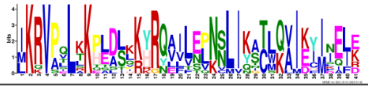
6	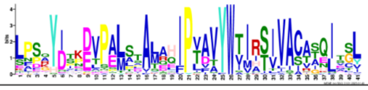
7	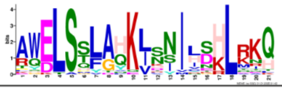
8	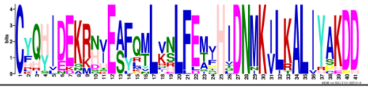
9	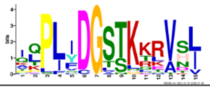
10	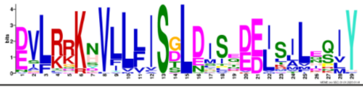
11	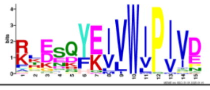
12	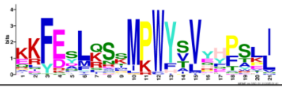
13	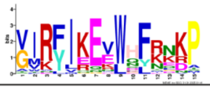
14	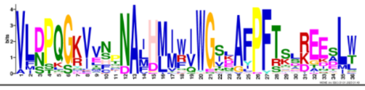
15	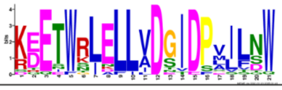
16	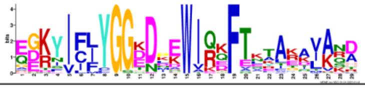
17	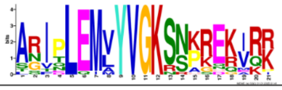
18	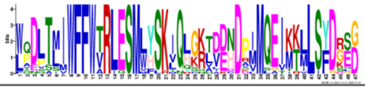
19	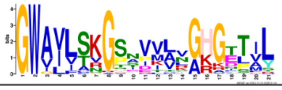
20	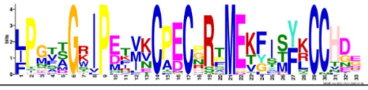
